# Carbon Nanomaterials Modified Biomimetic Dental Implants for Diabetic Patients

**DOI:** 10.3390/nano11112977

**Published:** 2021-11-05

**Authors:** Renjini Vijay, Jayanti Mendhi, Karthika Prasad, Yin Xiao, Jennifer MacLeod, Kostya (Ken) Ostrikov, Yinghong Zhou

**Affiliations:** 1 School of Mechanical, Medical and Process Engineering, Faculty of Engineering, Queensland University of Technology (QUT), Brisbane, QLD 4000, Australia; renjini.vijay@hdr.qut.edu.au (R.V.); jayantiarun.mendhi@hdr.qut.edu.au (J.M.); karthikaprasad@live.com (K.P.); yin.xiao@qut.edu.au (Y.X.); 2 Centre for Biomedical Technologies, Queensland University of Technology (QUT), Brisbane, QLD 4000, Australia; kostya.ostrikov@qut.edu.au; 3 School of Engineering, College of Engineering and Computer Science, Australian National University, Canberra, ACT 2600, Australia; 4 The Australia-China Centre for Tissue Engineering and Regenerative Medicine (ACCTERM), Queensland University of Technology (QUT), Brisbane, QLD 4000, Australia; 5 School of Chemistry and Physics, Faculty of Science, Queensland University of Technology (QUT), Brisbane, QLD 4000, Australia; jennifer.macleod@qut.edu.au; 6 Centre for Materials Science, Queensland University of Technology (QUT), Brisbane, QLD 4000, Australia

**Keywords:** biomimetic, dental implants, carbon, nanomaterials, diabetes mellitus

## Abstract

Dental implants are used broadly in dental clinics as the most natural-looking restoration option for replacing missing or highly diseased teeth. However, dental implant failure is a crucial issue for diabetic patients in need of dentition restoration, particularly when a lack of osseointegration and immunoregulatory incompetency occur during the healing phase, resulting in infection and fibrous encapsulation. Bio-inspired or biomimetic materials, which can mimic the characteristics of natural elements, are being investigated for use in the implant industry. This review discusses different biomimetic dental implants in terms of structural changes that enable antibacterial properties, drug delivery, immunomodulation, and osseointegration. We subsequently summarize the modification of dental implants for diabetes patients utilizing carbon nanomaterials, which have been recently found to improve the characteristics of biomimetic dental implants, including through antibacterial and anti-inflammatory capabilities, and by offering drug delivery properties that are essential for the success of dental implants.

## 1. Introduction

**Figure 1:**
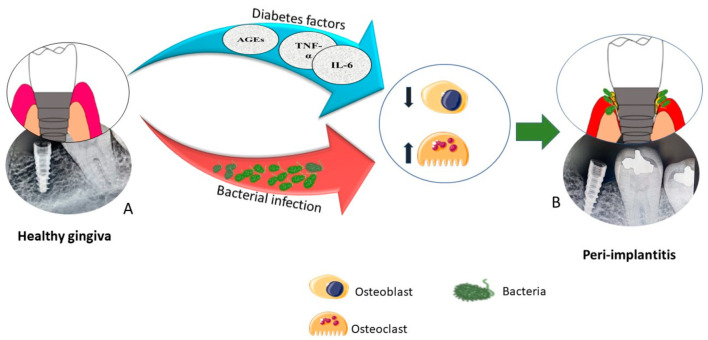
Effect of diabetes mellitus on dental implant failure. (**A**) Illustration and a representative intra oral periapical image (IOPA) of a diabetic patient who had received implant placement. This was taken immediately after the surgical placement of a dental implant. (**B**) Illustration and a follow up IOPA showing the failure of the dental implant due to infection. Image courtesy of Dr. Bijoy John, Indira Gandhi Institute of Dental Science, Kerala, India.

Dental implants, which can be used to replace one or more lost teeth or to anchor dentures in the mouth, are an effective alternative to traditional dentures or bridgework for the restoration of missing teeth [1]. The success of osseointegration, the direct interface formed between implant and bone, with no intervention of soft tissues after implantation is crucial to its long-term survival [2,3,4,5]. Any disruption in this biological mechanism could have a detrimental effect on the therapy outcome [2]. Diabetes mellitus (DM), for example, can contribute to dental implant failure, as uncontrolled diabetes is associated with a tendency to develop peri-implantitis and poor osseointegration [2,6]. Studies show a strong correlation between diabetic patients and the incidence of peri-implant infection [6,7,8,9], as well as reduced osseointegration [10], as impaired glycemic control increases the release of toxic metabolites (advanced glycation end products AGEs) [11], and inflammatory cytokines interleukin 6 (IL-6) and tumor necrosis factor-α (TNF-α), which lead to a slowed healing process for dental implants, and subsequent infection and implant failure (Figure 1) [12,13]. Therefore, the need to develop novel materials to enhance osseointegration, especially in diabetic patients, is clearly established.

Biomimetics is a relatively new discipline that uses notions from nature to create superior materials for use in a variety of applications, including healthcare devices [14,15]. By applying the lessons learned from the development and function of naturally occurring materials or structures, similar products have been synthesized to mimic their distinctive properties [16]. Since the early 1990s, the emphasis on nanoscience and nanotechnology has spurred interest in replicating nature through nanofabrication techniques for commercial purposes [17], including dentistry where biomimetics aims to restore the deformed dentition [14]. Dental implants with biomimetic characteristics improve osseointegration efficiency, and there are a number of examples from nature that are inspiring the way the dental implants are developed. Cicada wings, for example, have nanopatterns with antibacterial qualities [18,19,20], whereas other nature-inspired nanopatterns aid in the achievement of self-cleaning [21] and antibacterial properties [19], and will be examined in detail later. Cicada-inspired biomimetic materials do deliver better results than traditional implants though they are yet to be developed so as to provide antimicrobial action against *Staphylococcus aureus* [19], bacteria that can affect the immune response and cause peri-implant bone loss during the early stages of dental implant recovery [22,23]. Teixeira-Santos and colleagues established that a dental implant coated with carbon nanotubes, a carbon nanomaterial, have antibacterial characteristics against *Staphylococcus aureus* [23], which is significant because the size of a material effects its antibacterial properties [24]. Carbon nanotubes in some studies have been reported with a diameter of 0.7 nm [25,26], whereas cicada inspired materials have cone-shaped nanopillars with a diameter of 82–148 nm [27,28]. The surface area of a material rises as its size reduces, enhancing the substance’s ability to interact with and be taken up by living cells [29], which means the structure and properties of the materials used for modifying the implants play an important role in the success and longevity of a dental implant.

The unique chemical and physical characteristics, namely thermal, mechanical, electrical, optical, and structural features of carbon-based nanomaterials have sparked the interest of researchers for biological applications [30,31], and make them suitable for various dental applications (Table 1). Carbon-based nanoparticles like carbon nanodots, nano diamonds, graphene, and carbon nanotubes are applied broadly in many biomedical fields and for applications such as nanotherapeutics, biomedical imaging, and cancer therapy [32]. This review addresses the use of carbon in the development of dental materials, particularly biomimetic dental implants functionalized with carbon nanomaterials for local insulin delivery [33] to achieve antibacterial and osteogenesis properties for improved dental implant survival rates in diabetic patients [34].

## 2. Biomimetic Dental Implants

Biomimetic dental implants can be achieved by modifying the surface of an implant material [51]. Surface properties such as surface roughness are important factors governing the success of dental implants [52], and it is the intrinsically nanostructured surface features of the cicada [53,54,55], dragonfly [56,57], shark skin [57,58], gecko feet [21], taro [59,60,61], and lotus leaves [62,63] that are able to impart self-cleaning and antibacterial capabilities to dental implants [19]. Biomimetic dental implants have many advantages over ordinary implants; for example, modified surfaces or functionalized biomimetic dental implants can help increase antimicrobial action [19] by adding a functional group with antimicrobial properties [51]. Roughened surface structures have increased surface areas and can influence cell growth and differentiation, antimicrobial properties, and also improve specific protein interactions [64].

### 2.1. Bio-Inspired Patterns Used to Increase Antimicrobial and Antifouling Properties

Bacterial biofilm development on the transmucosal component of dental implants can induce persistent inflammation and implant failure [65,66]. The presence of high glucose levels in the saliva and blood as seen in diabetic patients induces poor neutrophil activity, tiny vessel damage, and neuropathy, further promoting bacterial growth and colonization [67,68,69]. *Staphylococcus aureus*, *Enteric bacilli*, and *Candida albicans*, as well as *Prevotella nigrescens*, *Campylobacter rectus*, *Campylobacter rectus*, and *Aggregatibacter actinomycetemcomitans*, particularly serogroup B, are the major species of bacteria resulting in peri-implantitis [70]. Infection plays a major role in peri-implantitis and the failure of dental implants for those with DM. Although the administration of post-operative antibiotics as well as an oral antimicrobial rinse can control peri-implantitis for uncontrolled diabetic patients [71], there is a lack of understanding about the choice and effectiveness of systemic antibiotic drug delivery [72,73], despite the number of preventive systemic antibiotic regimens proposed to reduce infections following dental implant implantation [74].

#### 2.1.1. Insect-Inspired Patterns

Systemic antibiotic applications have drawbacks such as systemic toxicity [75], low bioavailability, and antibiotic resistance [74,76,77], making local antibiotic treatment a preferred method of drug administration [76]. Studies have shown many potential benefits from localized drug delivery for peri-implantitis [77,78,79]. Some of the localized drug delivery approaches applied in implants are the surface immobilization technique [80], controlled release of a drug from coated implants [81], and dental implants with internal channels. The development of smart biomimetic dental implants enhanced with carbon nanomaterials (CNMs) marks a turning point for numerous applications and therapies, particularly with the precise and timely release of medication [82].

Several studies have attempted to mimic the nanotexture of naturally occurring surfaces such as cicada [83], dragonfly wings [56], and modified gecko feet [84], in order to modify dental implants by creating an antibacterial surface. These structurally altered superhydrophobic surfaces have proven particularly appealing as stable antibacterial surfaces because their self-cleaning and water-resistant characteristics prevent the development and adherence of bacteria rather than immediately destroying them [85]. These superhydrophobic materials can minimize the adhesion force between bacteria and the solid surface of implants, providing an antifouling effect on the surface [86]. The development of microorganisms resistant to antimicrobial therapies [87,88] has made it difficult to treat infection in diabetic patients [89], so it is anti-infective biomimetic materials that demonstrate bacteriostatic or bactericidal properties, along with a pro-osteogenesis function mediated by nanostructured surfaces [90] that have piqued the curiosity of researchers. The gecko is famous for its bulbous toes, covered with hundreds of microscopic hairs known as setae, and capable of adhering to different surfaces because of its periodic array of hierarchical microscale setae [91]. The length of setae are 30–130 µm, which are divided into hundreds of nanoscale spatulas with a diameter of 200–500 nm [92], creating a bactericidal property against gram-negative and gram-positive bacteria [19]. By incorporating this nanostructure to modify dental implants, it is possible to achieve implant surfaces with bactericidal [19], antifouling, and super-hydrophobic properties [93].

The wings of cicadas are made of spiky, cone-shaped nanopillars [94] and show antibacterial activity [19] that when fabricated by nano-imprint lithography [95] and reactive ion etching [96] present an interesting antibacterial mechanism. When bacteria fall on a cicada wing, they contact the tips of nanopillars and move downwards. The cone shaped nanopillars stretch their membranes as the bacteria move, eventually breaking the bacterial membranes and killing the bacteria. Bacteria with a less stiff membrane structure are quickly stretched and destroyed, but those with a rigid structure can withstand the nanopillars for a longer period [97]. Ge and colleagues proved this disadvantage of cicada-inspired nanostructures [83]. Another nanostructural antibacterial agent is fluorine-doped hydroxyapatite (FHA). Hydroxyapatite, an important component of normal bones and teeth, provides stiffness to the structure. The cicada-inspired FHA nanopatterned surface is effective against both gram-positive and gram-negative bacteria. Hydroxyapatite coatings help achieve better cellular proliferation as well as differentiation [98,99,100] and have proven beneficial for diabetic patients. For instance, a recent study reported that coating dental implants with nanostructured hydroxyapatite and silicon-based substitutes can improve osseointegration for the diabetic group [101].

**Figure 2:**
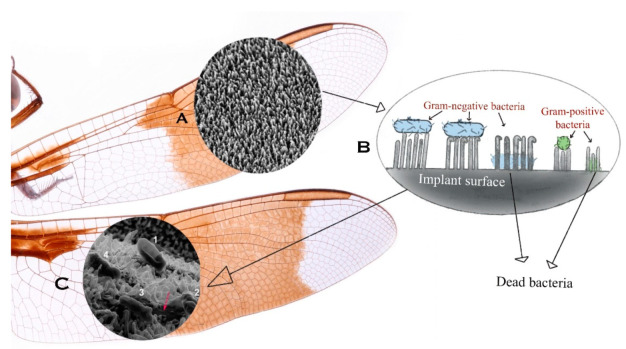
Schematic of dragonfly wings showing: (**A**) representative SEM image of nanopillar structures (magnified 20,000 times); (**B**) antibacterial mechanism of implants modified with dragonfly nanopillars; (**C**) representative SEM image showing nanopillar tearing the bacteria and causing the death of *Escherichia coli*. Adapted with permission [56].

Similarly, the dragonfly wing has a nanopillar structure [97]. The dragonfly surface is superhydrophobic with a 153° and above water contact angle [19,102]. Unlike the cicada, the protrusions are irregular and conical [58], and reported to be varying among different species with a range of 70 to 195 nm [19,103,104]. The dragonfly wing structures are active against both gram-positive and gram-negative bacteria [105] and exhibit both antibacterial and antifouling properties [106]. Hydrothermal synthesis [107] and reactive ion etching [93] are fabrication techniques that can produce effective antibacterial surfaces with the dragonfly pattern. Bhadra et al. generated hierarchically structured titanium nano-patterned arrays using hydrothermal synthesis followed by a high temperature treatment; these surfaces provided bactericidal effects and increased osseointegration [56]. The nano-wire arrays produced on titanium substrates were found to be comparable to the natural bactericidal nano-patterns seen on dragonfly wings [108]. Taller nanostructures begin to bend when gram-negative bacteria, such as *Escherichia coli*, are exposed to the nanostructure of dragonfly wings. Thereafter, bacterial cells adhere firmly to nanostructures because of the production of an extracellular polymeric substance (EPS) layer. Once the adhesion force is strong enough, the bacterial membranes break, causing the death of bacteria (Figure 2) [19]. Because it is effective for both gram-positive and gram-negative bacteria, this nanopattern will be a useful strategy for developing biomedical device surfaces.

#### 2.1.2. Animal-Inspired Patterns

Shark skin also exhibits antifouling properties [58], which can prevent bacterial adhesion [104]. Shark skin has a complex surface structure pattern of placoid scales or dermal denticles. These denticles have a riblet-like form, and the surface of the concave grooves feature nanostructured protuberances. Studies using the antifouling action of the shark skin show how riblet ridges on the surface change the water flow near the surface, helping to reduce drag on the body [109,110,111,112]. Previous research addressed other plausible pathways for antifouling abilities including epidermal mucus, which, like antimicrobial peptides, functions as a barrier for bacteria and hence has antibacterial properties. The microtopography of the shark skin prevents microbes from settling on the skin. Approaches for creating an artificial surface with shark skin characteristics include micro molding and vacuum casting [19]. Chien and colleagues recently reported that *Staphylococcus aureus* showed no surface attachment on a biomimetic shark skin pattern, but *Escherichia coli* demonstrated the reverse behavior in the early stages. Though *Escherichia coli* manifested surface attachment in the early stages, the pattern inhibited the biofilms from growing further [58].

#### 2.1.3. Plant-Inspired Patterns

The micro or nanopatterns on the leaves of lotus and taro plants with antifouling, super-hydrophobic, and self-cleaning characteristics are a growing source of inspiration and motivation for researchers to imitate their behaviors [113]. The micro elliptical bumps, coated with hierarchical waxy nanoscale epicuticular crystals 10–30 µm in diameter, make the surface hydrophobic. The antibacterial activity of lotus and taro leaves can also be attributed to a physicochemical interaction between the bacteria and the leaf surface where dirt and bacteria adhere to the water droplets rather than the surface [19].

### 2.2. Bio-Inspired Patterns Used to Increase Osseointegration

The effects of surface roughness on bacteria are controversial, with some stating that surface roughness helps in bacterial attachment and, others claiming the opposite [114,115,116]. Materials with increased surface roughness and surface free energy have higher super-hydrophobic characteristics [117], and it is the air entrapment phenomena on roughened surfaces that might explain this relationship [118]. When the roughened surface comes in contact with a fluid, the Cassie-Baxter state can be achieved, i.e., air is enclosed in the craters/valleys of the roughened surface [119]. The bacteria-containing medium should first wet the surface of the bacteria to promote bacterial adherence to the surface. Bacterial attachment is reduced when surface wetting of the bacteria is hindered by the hydrophobic surface by air entrapment during the first hour of contact [120]. Recent research has demonstrated that the mussel-inspired polydopamine (PDA) would help create surface roughened titanium implants [121] and improve osseointegration [122], which is essential for implant longevity in diabetes patients. Because of its exceptional characteristics, mussel-inspired PDA has emerged as a potential chemical for attaching synthetic and biological molecules or creating an adhesive layer onto different surfaces for biomedical and nanotechnology applications [123]. Mussels have a highly adhesive property, a result of mussel foot proteins released during sticky development in the mussel byssus adhesive plaque, which enables them to withstand the high shear stress of water flow. These foot proteins include 3,4-dihydroxy-L-phenylalanine (DOPA) and lysine amino acids, leading to the idea that the co-existence of catechol (DOPA) and amine (lysine) groups may be critical for generating robust adhesion [119]. PDA, which contains both catechol and amine groups [124] is used as a coating on various implant surfaces [125]. Su et al. used PDA and graphene oxide to coat titanium implants, which improved surface roughness, graphene oxide binding, cell survival, and osteogenic characteristics [126]. Another study on titanium implants used PDA as a coating to explain the creation of an efficient multifunctional surface that can fight off bacteria and support the reduction of oral biofilm-associated infections [66]. It is the mussel-inspired PDA that acts as a binding agent and aids the achievement of antibacterial characteristics while simultaneously promoting osseointegration. PDA also helps enhance the formation of hydroxyapatite and can modify nanoparticles used as drug carriers [127]. A recent study was conducted on a titanium implant covered with PDA to immobilize certain growth factors such as basic fibroblast growth factor (bFGF) and bone morphogenetic protein-2 (BMP-2) to promote cell migration, further accelerating wound healing and the formation of new bone around the implant [128]. Aside from bioinspired materials, several studies have been conducted to employ various materials to improve the osseointegration of dental implants (Table 2). Among these studies, Yang and colleagues reported that even under DM circumstances, the titanium dioxide nanotubes (TNT) surface showed good biocompatibility and pro-osteogenic activity in vitro and in vivo by producing less reactive oxygen species (ROS). The TNT surface offered a greater antioxidant ability by producing more total superoxide dismutase (SOD) to balance the ROS expression, therefore alleviating the inhibition of osteogenesis in high-glucose situations [129]. Additionally, gold nanoparticles coupled with microRNA 204 (miR204) inhibitor distributed in poly (lactic-co-glycolic acid) (PLGA) solution were employed in a recent study to boost the osteogenic capacity of bone mesenchymal stromal cells. The administration of anatgomiR204-conjugated gold nanoparticles restored miR204 misexpression and promoted osteogenesis in diabetic rats [130]. Similarly increasing the surface roughness of an implant can lead to increased osseointegration because of the increased area for cell attachment to the implant surface and can improve peri-implant bone wound healing [131].

## 3. The Delivery of Insulin to Improve Dental Implants for Diabetic Patients

Patients with poorly controlled diabetes have delayed osseointegration following implantation [10], whereas insulin plays a vital role in wound healing and the success of dental implants in diabetic patients [140]. In both diabetic and non-diabetic patients, the topical application of insulin in a sustained release manner helps improve wound healing by faster wound contraction and re-epithelization without affecting normal blood glucose levels [33,141,142] According to a recent study, 10 units of topical insulin are safe without changing blood glucose levels, suggesting its potential application in non-diabetic patients’ wounds. Furthermore, the study proposed that local insulin is acceptable for routine therapeutic usage in wound healing [143]. Kaur et al. discovered that silver nanoparticles with a sustained release of 50 μL insulin encouraged wound healing by regulating the balance of inflammatory cytokines at the wound site [144]. The systemic application of insulin can lead to an increased formation of granulation tissues and new vessels [33] with a similar result seen with the topical application of insulin on titanium discs. Malekzadach et al. coated the titanium discs with insulin to investigate the release of insulin immobilized on the discs, and the subsequent biological effects on the osteoblast-like cells. That study demonstrated the controlled release of insulin is beneficial for the mineralization process [145]. The significance of insulin release is evident from a diabetes experimental model showing a decreased amount of bone-implant contact, which was restored with insulin therapy. Insulin promotes the production of the osteoblastic matrix directly and in diabetes experimental models, normoglycemia levels achieved by insulin therapy resulted in bone matrix development and osteoid production comparable to the control subjects. In order to achieve a better outcome with biomimetic dental implants for diabetic individuals, insulin delivery can be very beneficial [146].

## 4. Capabilities of Carbon Nanomaterials to Functionalize Biomimetic Implants

### 4.1. Drug Delivery Property

Carbon nanomaterials (CNMs) have unique features that make them one of the most promising nanomaterials in dentistry and are used as drugs themselves or gene carriers [147,148]. For example, graphene has been applied as a nano drug with a high pharmaceutical efficiency, and low toxicity of the CNMs and anti-inflammatory properties have been achieved by combining CNMs with different drugs, proteins, nucleic acids, and bioactive peptides [138].

**Figure 3:**
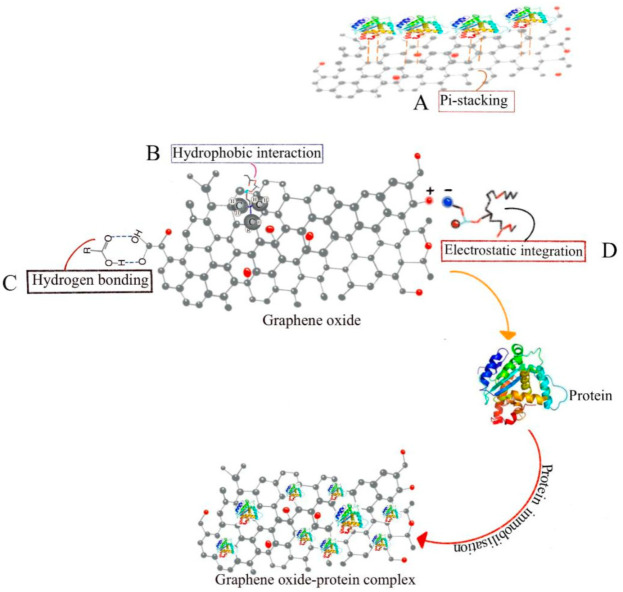
Illustrating different non-covalent bonds in protein binding. (**A**) Pi-stacking noncovalent interaction helps proteins bind to graphene oxide; (**B**) Hydrophobic interaction helps increase protein stability and bioactivity; (**C**) Hydrogen bonding stabilizes the protein structure; (**D**) Electrostatic integration is important in protein folding, stability, flexibility, and function.

CNMs carry drugs through the π-π (Pi) stacking interaction [149]. A variety of drugs have been delivered through π-π stacking interactions, methods that have been utilized in biological medication delivery, such as protein, nucleic acid, and cell delivery [150,151,152]. These π-π stacking interactions are non-covalent, do not change the structural or functional characteristics of medicines, and have been employed as a driving factor in loading medications into delivery systems and the creation of self-assembling systems [153]. Noncovalent interactions (Figure 3) such as hydrogen bonding [154], van der Waals force, and electrostatic, hydrophobic, or π-π interactions can physically confine drug molecules in delivery devices. CNMs coatings on biomimetic dental implants can therefore serve as a drug-releasing system [122], which eventually helps deliver insulin to the wound site, accelerating wound healing and osseointegration.

### 4.2. Antibacterial Property

Apart from their potential to serve as efficient drug delivery platforms, CNMs are also established antibacterial agents, most commonly via contact-mediated biocidal action [76]. The bacterial cell wall permeability is altered by the nanoparticles that are positively charged, which electrostatically attract the negatively charged bacterial cell membrane, resulting in its rupture, followed by leakage of intracellular organelles. Application of carbon nanoparticles against multidrug-resistant organisms is evolving, as they can disrupt the microbial membrane and its metabolic procedure more effectively than most other antibacterial materials used [155,156,157]. Drug carrying properties are achieved by the small particle size and high surface-to-volume ratio of the material, which is more effective in a nanocomposite form and not so by the antibiotic mechanisms, as is seen in conventional antimicrobial strategies [156,158]. For instance, nanocomposites and hybrid materials of Ag-metal-organic frameworks (MOFs) with carbon quantum dots (CQDs) have shown enhanced antibacterial activity against representative gram-positive (*Bacillus subtilis*) and gram-negative (*Escherichia coli*) bacterial strains due to nanorod-like morphological features and specific surface chemistry [158]. Carbon quantum dots are small carbon nanoparticles less than 10 nm in size [159,160,161]. Additionally, graphene nanocomposites have a greater ability against gram-negative and gram-positive bacteria, which is achieved by the ability of graphene to penetrate and cut the cell membrane, thereby damaging the bacteria [162]. Therefore, the coating of biomimetic dental implants with CNMs could be a promising strategy to prevent implant failure.

### 4.3. Anti-Inflammatory Property

**Figure 4:**
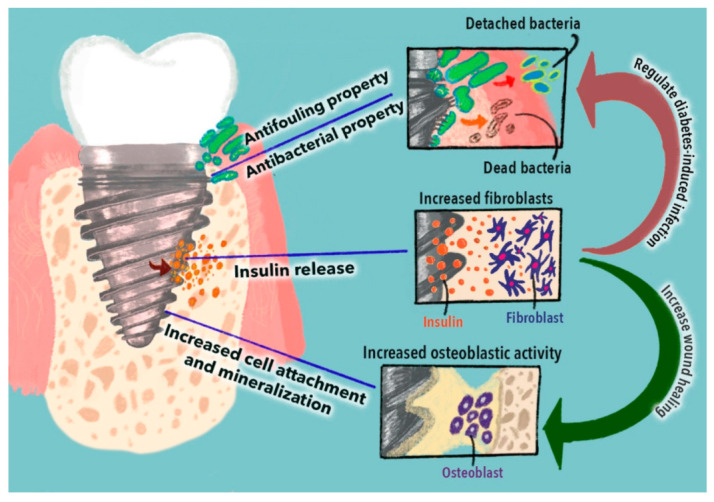
Strategies to modify biomimetic dental implants for diabetic patients.

The anti-inflammatory effects of dental implants are critical to their success in diabetic patients because peri-implantitis is the result of hyperglycemia-mediated inflammatory reaction [163]. CNMs have anti-inflammatory properties that can regulate immune cell activity and reduce the secretion of proinflammatory cytokines [164], and along with their large surface area and biocompatibility, help in their use as anti-inflammatory agent carriers [164]. A recent study evaluated the effects of graphene oxide (GO)-coated titanium surfaces on immune cell reaction and the subsequent osteogenesis of mesenchymal cells [126]. This study showed promising results in the immunoregulatory effect on osteogenesis and biocompatibility. In particular, there was an increase in the gene expression levels of osteogenic markers on the GO-coated titanium surface [126]. Therefore, it is postulated that functionalizing biomimetic dental implants with carbon nanomaterials aids in the prevention of implant failure and increases the success rate in diabetic patients (Figure 4).

## 5. Conclusions

The use of biomimetic dental implants may improve the success rate of implants for diabetic patients, and their antibacterial properties may assist with the prevention of peri-implantitis. The use of carbon nanostructures in drug delivery systems may enable these materials to efficiently carry drugs and mimic protein channels, helping to achieve a better result in wound healing and thereby reduce the failure rate of dental implants. It is the adaptability of nanomaterials, particularly with carbon nanomaterials for modifying biomimetic dental implants that can address issues like infections, delayed wound healing and osseointegration, create long-lasting dental implants, and contribute to the development of personalized dental therapy for diabetic and non-diabetic patients with delayed wound healing.

## Figures and Tables

**Table 1:** Application of carbon nanoparticles in dentistry.

Carbon Nanoparticles	Dental Applications
Carbon nanodots	Dental robots for prevention of caries and gingival diseases [35]
Nano diamonds	Root canal as gutta percha [36] Surface modification in dental implants [37] Drug delivery system [38] Oral cancer treatment [39] Dental materials [40]
Graphene	Implants [41] Guided bone regeneration [42] Resins and cements [43] Teeth whitening [44] Electronic sensors [45]
Carbon nanotubes	Drug delivery system [46] Alveolar ridge augmentation [47] Screws and plates [48] Guided bone regeneration [49] Dental implants [50]

**Table 2:** Different materials used for dental implant surface coating to improve osteointegration.

Type of Materials	Advantage as Dental Implant Coating on Osseointegration
Titanium oxide nanotubes coated surface	Enhance implant osseointegration and inhibit osteoclast formation by activating bone cell viability in vitro [132]
Hydroxyapatite-coated surface	A significant increase in the amount of new bone growth, bone-to-implant contact, and surface roughness [133]
Hydroxyapatite and silicon-based coating	The coating modified by electrochemical method has been reported to have potentially beneficial chemical and physical properties that promote osteointegration by increasing the bone to implant contact [134]
Chitosan gold nanoparticle coating	Promote osseointegration of dental implants even in osteoporotic conditions [135]
Laser-deposited titanium dioxide nanoparticles	Significant improvement in bone integration, surface roughness values, and binding strength at the bone-implant interface [136]
Aluminium oxide nanoparticles	Promote the formation of new bone with Haversian canals, osteoblasts, and osteocytes [137]
Polydopamine coated dental implants	Helps in functionalising the implant material and increases the attachment of the bone cells and helps in wound healing [129,138]
Gold nanoparticles coupled with miR204	Restores miR204 misexpression, and increases the osteogenic activity of bone mesenchymal stromal cells [130]
Calcium phosphate	Accelerate implant fixation and bone healing response by increased bone-to-implant contact [139]
